# A combination of valproic acid sodium salt, CHIR99021, E-616452, tranylcypromine, and 3-Deazaneplanocin A causes stem cell-like characteristics in cancer cells

**DOI:** 10.18632/oncotarget.18396

**Published:** 2017-06-07

**Authors:** Shuang Sha, Yuanfen Zhai, Chengzhao Lin, Heyong Wang, Qing Chang, Shuang Song, Mingqiang Ren, Gentao Liu

**Affiliations:** ^1^ Tongji University School of Life Sciences and Technology, Shanghai, China; ^2^ Clinical Research Center, Jiading District Central Hospital Affiliated Shanghai University of Medicine & Health Sciences, Shanghai, China; ^3^ Department of Immunity, Tongji University School of Medicine, Shanghai, China; ^4^ Center for Translational Medicine, Shanghai Pulmonary Hospital, Tongji University School of Medicine, Shanghai, China; ^5^ Center for Cancer Immunotherapy, Shanghai Biomed-Union Biotechnology Co. Ltd, Shanghai International Medical Zone, Shanghai, China

**Keywords:** non-small cell lung cancer, lung cancer stem-like cells, CD133, small molecule compounds, NOTCH signaling

## Abstract

Many studies are based on the hypothesis that recurrence and drug resistance in lung carcinoma are due to a subpopulation of cancer stem-like cells (CSLCs) in solid tumors. Therefore it is crucial to screen for and recognize lung CSLCs. In this study, we stimulated non-small cell lung cancer (NSCLC) A549 cells to display stem cell-like characteristics using a combination of five small molecule compounds. The putative A549 stem cells activated an important CSLC marker, CD133 protein, as well multiple CSLC-related genes including ATP-binding cassette transporter G2 (ABCG2), C-X-C chemokine receptor type 4 (CXCR4), NESTIN, and BMI1. The A549 stem-like cells displayed resistance to the chemotherapeutic drugs etoposide and cisplatin, epithelial-to-mesenchymal transition properties, and increased protein expression levels of NOTCH1 and Hes Family bHLH Transcription Factor 1 (HES1). When A549 cells were pretreated with a NOTCH signaling pathway inhibitor before compound induction, expression of the NOTCH1 target gene HES1 was reduced. This demonstrated that the NOTCH signaling pathway in the putative A549 stem-like cells had been activated. Together, the results of our study showed that a combination of five small molecule agents could transform A549 cells into putative stem-like cells, and that these compounds could also elevate CD133 and ABCG2 protein expression levels in H460 cells. This study provides a convenient method for obtaining lung CSLCs, which may be an effective strategy for developing lung carcinoma treatments.

## INTRODUCTION

Lung cancer is one of the most common malignant carcinomas, with a poor 5-year survival rate of about 15% [1]. Non-small cell lung cancer (NSCLC) accounts for approximately 80% of all lung carcinomas and has high mortality due to tumor growth, recurrence, and drug resistance. Many studies have found that a subpopulation of lung cancer cells called cancer stem-like cells (CSLCs) are correlated with recurrence and drug resistance, which has led to a cancer stem cell hypothesis [2, 3]. CSLCs have self-renewal and non-directional differentiation tendency capabilities [4]. Therefore, it is crucial to identify and isolate them in solid tumors to potentially improve cancer treatments and provide important predictive and prognostic information for lung cancer patients. However, since only a small number of CSLCs exist in each tumor, identifying and isolating them have proven challenging.

Many strategies have shown the potential to identify and isolate putative cancer stem cells. ATP-binding cassette transporters (ABCG2 and ABCB1) and multidrug-resistant protein 1 (MDR1) are important multi-drug resistant genes, which are also considered stem-like cell markers for many cancers. CSLC populations highly express ABC transporters, which can efflux the fluorescent dye Hochest33342 from cells. The dye-negative cells can be sorted by flow cytometry as a side population of cells that display cancer stem-like properties [5, 6]. Cisplatin and etoposide are common chemotherapeutic drugs used for lung cancer patients, and low concentrations of cisplatin can be used to identify CSLCs [7, 8]. CD133 (prominin-1) is a membrane glycoprotein that is a reliable CSLC marker for leukemia and ovarian, as well as for gastrointestinal and lung carcinoma [9–12]. CD133-positive tumor cells display many CSLC features, including involvement with cell sphere formation, and show high invasiveness and drug efflux [12, 13]. Therefore it can be used to identify CSLCs by cell sorting. Some researchers have transduced several transcription factors such as octamer-binding transcription factor 4 (OCT4), sex determining region Y-box 2 (SOX2), and kruppel-like factor 4 (KLF4) into tumor cells, which can enhance certain cell-stemness properties including self-renewal and chemo-resistance [14–17].

Several methods have been developed to generate tumor stem-like cells, but these methods have not been extensively applied and in many cases genetically modify tumor cells. Therefore, it is crucial to develop a reliable and convenient approach that can stimulate normal cancer cells into becoming putative cancer stem-like cells. Recently, small molecule compounds have been reported as a new method to induce mouse somatic cells to be reprogrammed to generate pluripotent stem cells [18]. This approach is simple, convenient, and does not require extensive materials. We hypothesized that small molecule compounds can also induce cancer cells to generate CSLCs. Valproic acid sodium salt is a sodium salt form of valproic acid (VPA), which is a histone deacetylase inhibitor with an IC_50_ of 0.4 mM and can stimulate hematopoietic stem cell proliferation and self-renewal [19]. CHIR99021 is a glycogen synthase kinase 3α (GSK-3α) and GSK-3β inhibitor with IC_50_ values of 10 and 6.7 nM, respectively [20]. E-616452 is a TGF-β receptor I kinase/activin-like kinase 5 (ALK5) inhibitor with IC_50_ values of 4 and 23 nM for TGF-β type I receptor autophosphorylation and binding, respectively [21]. 3-Deazaneplanocin A (DZnep) is an anti-metastatic agent that inhibits S-adenosylmethionine-dependent methyltransferase [22, 23]. These small molecule compounds can induce mouse somatic cells to become pluripotent stem cells. However, whether small molecule inhibitors can induce common tumor cells to become putative tumor stem-like cells has not been thoroughly studied.

Thus, the goal of this study was to investigate if small molecule inhibitors can stimulate A549 cells into becoming putative A549 stem-like cells. We found that treatment with a mixture of five small molecules (VPA sodium salt, CHIR99021, E-616452, tranylcypromine, and DZnep) could induce A549 cells to become putative tumor stem-like cells. We hope this approach can be applied to other types of tumor cells in future studies.

## RESULTS

### Morphology and growth of A549 cells after exposure to small chemical agents

**Figure 1:**
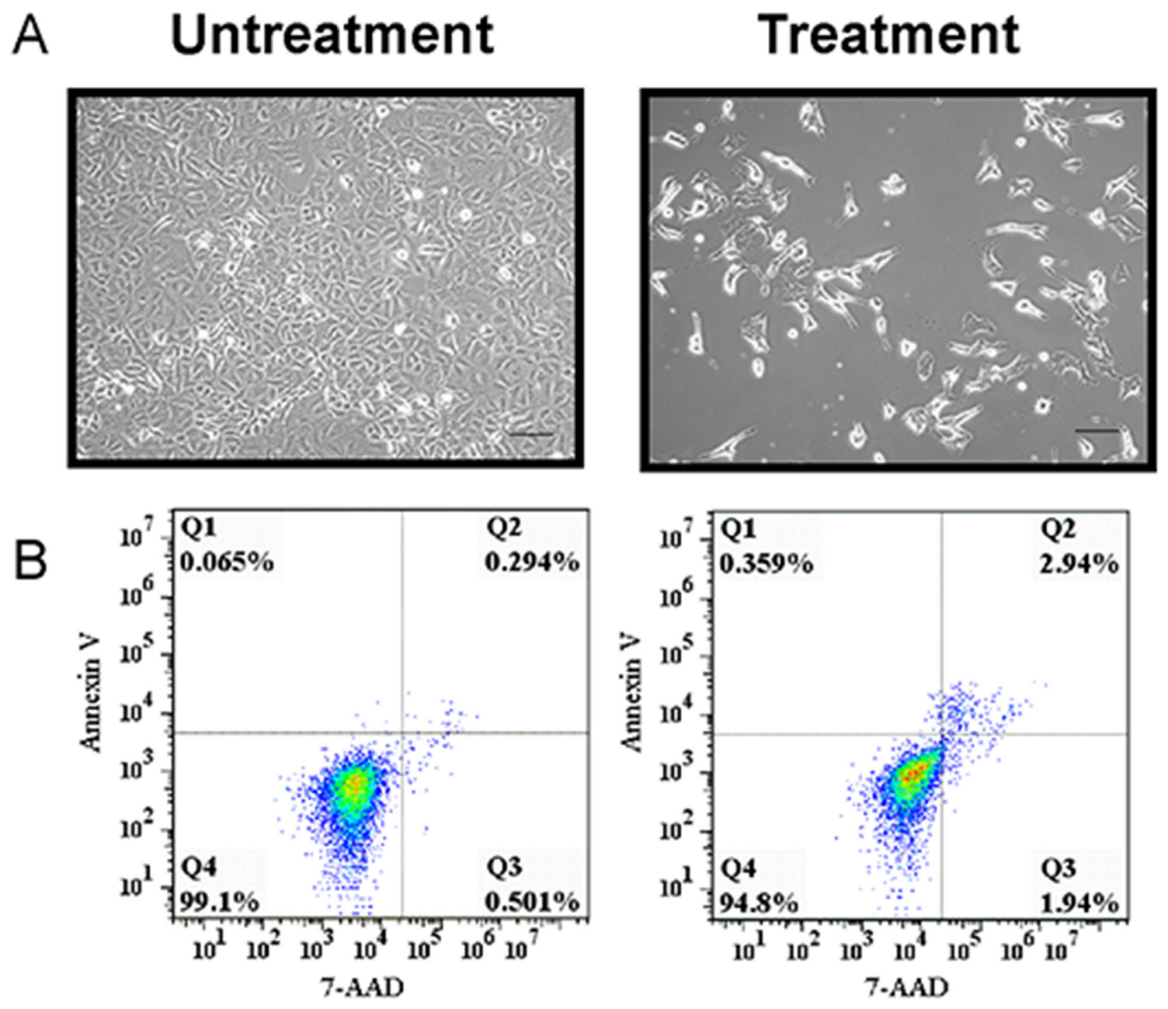
Representative images and cell viability of putative cancer stem cells and A549 cells **(A)** Representative images of putative A549 stem cells and control cells. **(B)** Putative A549 stem cells and A549 cells were stained with 7-AAD and Annexin V, respectively, and examined with flow cytometry.

To induce A549 cells to become tumor stem-like cells, we combined five small chemical compounds to treat the A549 cells, which have been shown to induce mouse fibroblasts to become pluripotent stem cells [18]. We hypothesized that the mixture of five compounds could stimulate a few A549 cells to become putative stem-like cells. After the A549 cells had been stimulated by the five small chemical compounds for 7 days, we examined the cell morphology and growth (Figure 1). From the morphology image results, we found that the morphology of the putative A549 stem cells had changed from a spindle to a slender shape and some cells were polygons, whereas the untreated cells were spindle shaped (Figure 1A). To evaluate cell growth, we used Annexin V and 7-AAD dyes to measure their apoptosis. We found that 0.065% and 0.359% of untreated and treated cells, respectively, were Annexin V-positive. We also found that 0.501% and 1.94% of untreated and treated cells, respectively, were 7-AAD-positive. Therefore, most of the treated cells did not display early or late apoptosis (Figure 1B). A total of 99.1% of the untreated cells and 94.8% of the treated cells were viable and alive, which was not statistically different. The growth of the remaining A549 cells did not exhibit apoptosis when treated with the small molecule agents, although more apoptosis would have been observed if the treatment time was extended.

### Putative A549 stem cells expressed CD133 and other tumor stem-like cell markers

**Figure 2:**
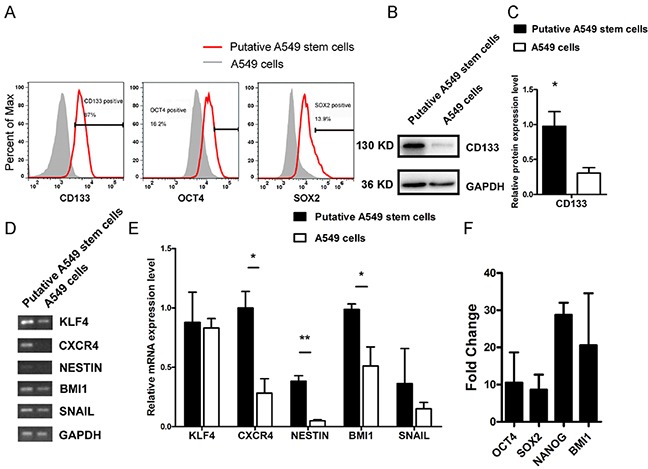
Relative stem-like features of putative A549 stem cells and A549 cells **(A)** CD133, OCT4, and SOX2 protein expression in putative A549 stem cells and A549 cells examined with flow cytometry. **(B)** Western blot results for CD133 protein in putative A549 stem cells and A549 cells. **(C)** Data analysis of CD133 expression. **(D–E)** sqRT-PCR analysis of KLF4, CXCR4, NESTIN, BMI1, and SNAIL gene expression levels in putative A549 stem cells and A549 cells. **P<0.01 **(F)** quantitative RT-PCR mRNA expression of OCT4, SOX2, NANOG, BMI1 in putative A549 stem cells.

CD133, OCT4, and SOX2 are cell markers for human CSLCs. To investigate the putative stem-like cell expression of these tumor stem cell markers, we used flow cytometry to examine CD133, OCT4, and SOX2 expression in putative A549 stem cells. We found that the putative A549 stem cells had 87% more CD133 expression than control cells, whereas SOX2 and OCT4 genes were stimulated by 13.9% and 16.2%, respectively (Figure 2A). CD133 has been generally acknowledged as a marker for many types of tumor stem-like cells, so we used Western blotting to investigate its protein expression (Figure 2B, 2C). We found that there was almost threefold more CD133 protein expression in putative A549 stem cells than in untreated cells. Thus, simultaneous treatment with the five small chemical compounds had the ability to stimulate normal A549 cells to become putative A549 stem cells. To confirm the results, we analyzed the relative mRNA expression levels of KLF4, C-X-C chemokine receptor type 4 (CXCR4), NESTIN, BMI1, and SNAIL in the putative A549 stem cells (Figure 2D, 2E). Results of sqRT-PCR showed that CXCR4, NESTIN, and BMI1 displayed significant differences compared with A549 control cells, whereas KLF4 and SNAIL gene expression showed no significant difference between the control and putative A549 stem cells (Figure 2D, 2E). Other genes such as OCT4, SOX2, NANOG, and BMI1 were stimulated, as determined by qRT-PCR (Figure 2F). The results demonstrate that putative A549 stem cells express tumor stem-like cell-related genes and may display tumor stem cell characteristics.

### Putative A549 stem cells have chemotherapeutic drug resistance characteristics

**Figure 3:**
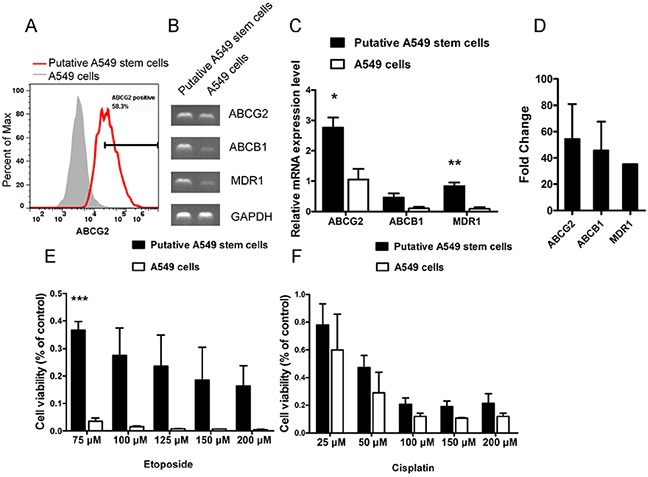
Cell viability of A549 cells treated with different concentrations of chemotherapeutic drugs for 48 h **(A)** Putative A549 stem cells stained with ABCG2-PE antibody and examined with flow cytometry. **(B–C)** mRNA expression of ABCG2, ABCB1, and MDR1 in putative A549 cells. **(D)** Quantitative RT-PCR results of ABCG2, ABCB1, and MDR1 in putative A549 stem cells. **(E)** Cell viability analysis of putative A549 stem cells and A549 cells treated with different concentrations of etoposide for 48 h. (75, 100, 125, 150 and 200 μM). **(F)** Cell viability analysis of putative A549 stem cells and A549 cells treated with different concentrations of cisplatin for 48 h. (25, 50, 100, 150 and 200 μM).

Many types of tumor stem-like cells display chemotherapeutic drug resistance, which is the mechanism underlying tumor recurrence. To analyze whether the putative A549 stem cells had chemotherapeutic drug resistance characteristics, we analyzed the expression levels of the common multi-drug resistance markers MDR1, ABCB1 and ABCG2. The flow cytometry results showed that ABCG2 protein expression was stimulated by 58.3% (Figure 3A). The putative A549 stem cells exhibited significantly higher expression levels of the multi-drug resistance markers ABCG2, ABCB1, and MDR1 compared with the control cells when analyzed by sqRT-PCR (Figure 3B–3D). These data show that the putative A549 stem cells had drug resistance characteristics. We also analyzed the resistance abilities of putative A549 stem cells to two commonly used drugs. Putative A549 stem cells were treated with common chemotherapeutic drugs used to treat NSCLC (etoposide and cisplatin), because etoposide and cisplatin are two systemic chemotherapy drugs for NSCLC [24]. The putative A549 stem cells were co-cultured with various concentrations of etoposide for 48 h, after which cell viability was measured using the CCK-8 assay. The putative A549 stem cells showed significant differences upon treatment with 75 μM etoposide (Figure 3E). We also studied the cytotoxic effects of the DNA-binding agent cisplatin on the putative A549 stem cells. To this end, putative A549 stem cells were treated with various concentrations of cisplatin for 48 h (Figure 3F). The IC_50_ of etoposide in putative A549 stem cells was 45.57 μM and that in control cells was 20.42 μM. The IC_50_ of cisplatin in putative A549 stem cells was 50.68 μM and that in A549 cells was 30.6 μM.

### Putative A549 stem cells have epithelial-to-mesenchymal transition and invasion phenotypes

**Figure 4:**
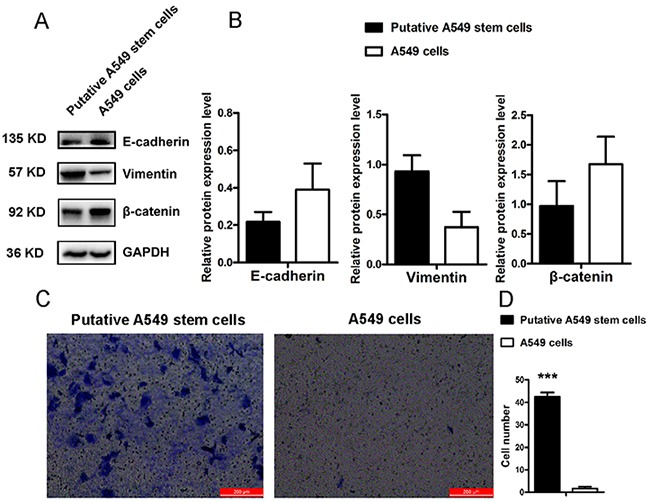
EMT features of putative A549 stem cells and A549 cells **(A–B)** Western blot results of E-cadherin, vimentin and β-catenin proteins in putative A549 stem cells and A549 cells. **(C–D)** Transwell invasion assay for putative A549 stem cells and A549 cells (***P<0.001).

Previous studies have confirmed that CD133^+^ lung cancer cells display chemical drug resistance and high tumorigenicity characteristics [13]. To investigate the invasive characteristics of putative A549 stem cells, we analyzed an important cancer stem cell property: epithelial-to-mesenchymal transition (EMT). We found that putative A549 stem cells also displayed EMT properties. Figure 4A shows the Western blot results for putative A549 stem cells expressing the EMT-related proteins E-cadherin, vimentin, and β-catenin. The expression of vimentin protein in putative A549 stem cells increased, while the expression of β-catenin and E-cadherin proteins decreased (Figure 4A, 4B). These results were in accordance with the EMT phenotype [25]. Studies have shown that EMT plays an important role in tumor metastasis [26]. To investigate the invasive capability of putative A549 stem cells, we analyzed the invasive capability of putative A549 stem cells (Figure 4C). Putative A549 stem cells showed a high degree of invasion capability compared with the control cells, and the number of invasive cells was significantly different (Figure 4D). The findings demonstrated that putative A549 stem cells isolated by compounds had a high invasion capability, while A549 cells could not migrate to the transwell bottom membrane.

### The NOTCH signaling pathway was activated in putative A549 stem cells

**Figure 5:**
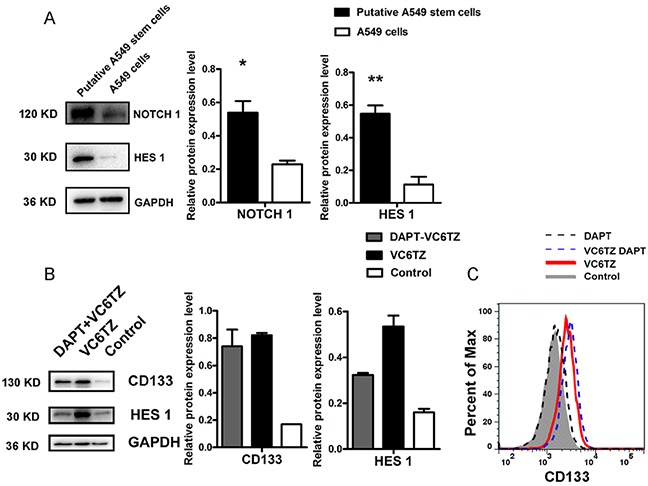
Relative expression of proteins in the NOTCH signaling pathway in putative A549 stem cells **(A)** Western blot results for NOTCH1 and HES1 expression levels in putative A549 stem cells and A549 cells. **(B)** Western blot results of CD133 and HES1 proteins in putative A549 stem cells and in cells pretreated with DAPT agent. **(C)** Flow cytometry results for CD133 expression in putative A549 stem cells and A549 cells after treatment with DAPT.

From the aforementioned results, we confirmed that putative A549 stem cells had stem cell gene CD133 stimulation and MDR characteristics. However, it has remained unclear which signaling pathway is activated in these cells. Studies have found that NOTCH signaling regulates cell polarity, cell-cell connection, and cell fate during cell development [27]. Some studies have suggested that the NOTCH signaling pathway may maintain tumor stem cell resistance and the EMT phenotype [28]. Therefore, we analyzed whether MDR characteristics and EMT phenomena of putative A549 stem cells associate with the NOTCH signaling pathway by analyzing the putative A549 stem cell NOTCH signaling pathway. Western blot analysis confirmed that NOTCH1 and HES1 protein expression in putative A549 stem cells was two-fold higher than that in A549 cells (Figure 5A). The NOTCH signaling pathway occurs via ligand protein binding to the extracellular domain, inducing proteolytic cleavage and release of the intracellular domain (NICD), which enters the cell nucleus to modify gene expression. A γ-secretase inhibitor DAPT, was used to inhibit the NOTCH signaling pathway. To analyze the effects of five small compounds on this pathway, we used 10 μM DAPT to pretreat A549 cells for 60 min, after which the cells were treated with the five agents. The CD133 expression of A549 cells pretreated with DAPT and then treated with the VC6TZ compounds (VPA sodium salt, CHIR99021, E-616452, tranylcypromine, and DZnep) was the same as the expression of the putative A549 stem cells. The Western blot and flow cytometry results showed that CD133 protein expression did not notably change (Figure 5B, 5C). For A549 cells treated with DAPT inhibitor alone, CD133 expression was the same as that found in the control cells (Figure 5C). Therefore, the inhibitor DAPT did not affect CD133 expression of putative A549 stem cells. By contrast, expression of the NOTCH signaling pathway downstream target gene HES1 decreased in putative A549 stem cells pretreated with DAPT (Figure 5B). The results showed that the NOTCH pathway had been activated in putative A549 stem cells, but CD133 expression was not regulated by the NOTCH signaling pathway in putative A549 stem cells.

### Relative protein expression of A549 cells after treatment with individual compounds

**Figure 6:**
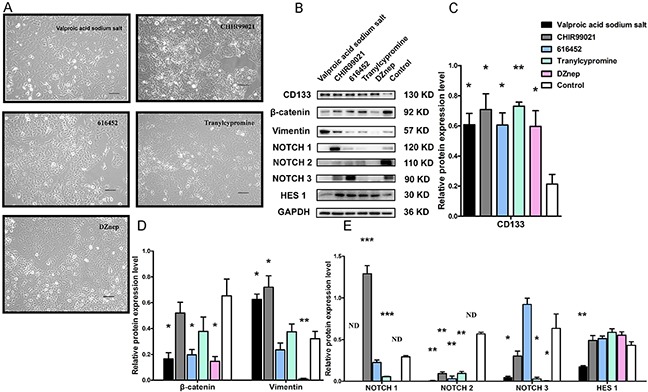
Relative protein expression in A549 cells with individual compound treatments **(A)** Representative images of A549 cells under treatment with VPA sodium salt, CHIR99021, E-616452, tranylcypromine and DZnep. **(B)** Western blot results show CD133, β-catenin, vimentin, NOTCH1, NOTCH2, NOTCH3 and HES1 expression in A549 cells after treatment with VPA sodium salt, CHIR99021, E-616452, tranylcypromine and DZnep, respectively. **(C–E)** Statistical data analysis of the Western blot results. (*** p<0.001) N.D.: no detection.

Putative A549 stem cell enrichment after treatment with a five small molecule compound combination increased CD133 expression and cell invasion capability. However, the mechanism by which each compound exerts its effects has not been well studied. To investigate the mechanism by which the small molecule chemical compounds affected the putative A549 stem cells, we used Western blotting to examine the expression of CD133, β-catenin, vimentin, NOTCH1, NOTCH2, NOTCH3, and HES1 after treatment of A549 cells with each compound individually (Figure 6B–6E). A subset of A549 cells was apoptotic after treatment and the remaining cells had no remarkable morphologic variations (Figure 6A). Western blot analysis showed that CD133 protein expression increased, and these results were statistically significant, especially for tranylcypromine treatment (Figure 6B, 6C). During EMT after treatment with the individual compounds, vimentin was stimulated by the VPA sodium salt, CHIR99021, but was decreased by DZnep treatment (Figure 6D). Treatment with the individual compounds decreased β-catenin protein expression, with the VPA sodium salt, E-616452, and DZnep agents having the most pronounced effects (Figure 6D). We also found that NOTCH1 protein was significantly increased by treatment with CHIR99021, but was significantly decreased by tranylcypromine. The NOTCH1 protein was no expression by the treatment with VPA sodium salt and DZnep, respectively (Figure 6E). NOTCH2 protein expression was decreased upon treatment with the individual compounds. VPA sodium salt, tranylcypromine, and DZnep agents decreased NOTCH3 protein expression (Figure 6E). The VPA sodium salt compound significantly decreased HES1 expression (Figure 6E).

## DISCUSSION

In this study, we used five small molecule chemical compounds (VPA sodium salt, CHIR99021, E-616452, tranylcypromine and DZnep) in combination to stimulate A549 cells so they would exhibit putative stem-like cell properties. The inhibitors led to the apoptosis of many normal A549 cells, but the surviving cells showed great promise as putative stem-like cells.

Recently, many types of CSLC markers have been identified including CD133, CD44, CD166, and ALDH for CSC [29]. Our results confirmed that the combination of five small molecule compounds enhances CD133 levels in putative A549 stem cells (Figure 2B). We also found that CD44, CD166, and ALDH markers were overexpressed in both A549 cells and putative stem-like cells (Supplementary Figure 1). In addition, some critical markers of tumor stem cells such as OCT4 and SOX2 transfactors were stimulated (Figure 2A, 2F). We also detected other cancer stem cell-related genes in the putative A549 stem cells, and found that CXCR4, NESTIN, BMI1 gene levels had increased (Figure 2D, 2E).

In order to investigate chemotherapeutic resistance ability of CSLCs, we detected the putative A549 stem cells’ resistant ability to cisplatin and etoposide. We found the putative A549 stem cells highly expressed the MDR genes ABCG2 and MDR1 (Figure 3A–3D) and were resistant to the chemotherapeutic agents etoposide and cisplatin (Figure 3E, 3F). Putative A549 stem cells did differ from the control A549 cells when treated with 75 μM of etoposide; however, the resistance ability of putative A549 stem cells to etoposide was higher than to cisplatin. These findings demonstrate that the putative A549 stem cells had MDR properties.

The EMT phenotype is increasingly recognized for its role in tumor invasion and metastasis [30]. Cells that undergo EMT are associated with decreased cell-to-cell adhesion, as well as increased motility and migration. Recently, the EMT phenotype has been found correlate with CSLC properties. Cells that have the EMT phenotype display vimentin gene stimulation, while the E-cadherin and β-catenin genes repress expression. CD133 is associated with the tumor EMT phenotype. We found that the putative A549 stem cells had the EMT phenotype and that levels of the EMT-related proteins β-catenin and E-cadherin decreased, while levels of vimentin increased. The invasion results (Figure 4C) showed that the invasive capability of putative A549 stem cells increased. VPA is a histone deacetylase inhibitor that can induce cell apoptosis, differentiation, and cell-cycle arrest. Previous studies have found that VPA promotes the EMT of colorectal cancer cells [31], although another research group indicated that VPA inhibited EMT in prostate carcinoma [32]. Our results demonstrated that VPA sodium salt promoted expression of the EMT marker vimentin protein in lung cancer cells (Figure 6B, 6D).

NOTCH is critical for maintaining cancer stem cell self-renewal and differentiation [33]. It is a transmembrane heterodimeric receptor in mammals, which involves four receptors (NOTCH1, 2, 3, and 4) and five ligands (Jagged1, Jagged2, Delta1, Delta3 and Delta-like4). The function of the NOTCH pathway in lung CSLCs remains unknown. We found that putative A549 stem cells displayed elevated NOTCH1 receptor expression levels, but did not show increased levels for the other NOTCH receptors. CHIR99021 is a GSK3β inhibitor that is a subtype of GSK-3. Previous studies have disagreed about the effects of GSK-3 on the NOTCH signaling pathway. Kim et al. [34] found that NOTCH signaling was enhanced in GSK-3 double-knockout mice, whereas another study [35] found that GSK-3β inhibition leads to decreased NICD levels in neuroblastoma cells and decreased vascular smooth-muscle cell proliferation and survival. In this study, when A549 cells were treated with CHIR99021 compound alone, NOTCH1 receptor expression increased. We found that CHIR99021 promotes NOTCH1 protein expression, while VPA sodium salt, tranylcypromine, and DZnep decreased NOTCH1 expression (Figure 6B). We also found that all five compounds decreased levels of NOTCH2. A previous study found that 5 μM CHIR99021 enhanced NOTCH3 expression in A549 cells during 24 h of treatment [36], but we found that 9 μM CHIR99021 did not increase NOTCH3 protein expression after treatment for 7 days (Figure 6B). Previous studies have found that VPA increased levels of the full-length NOTCH1 protein and transmembrane protein in neuroblastoma cells [37], as well as the NOTCH1 full-length protein and the intracellular domain in carcinoid cancer cells [38]. However, we found that 1 mM VPA sodium salt decreased transmembrane protein (NTM, approximately 120 kDa) levels (Figure 6B).

HES1 is one of the main genes targeted by NOTCH signaling and plays a critical role in maintaining the stemness of tumor stem cells. The tumor stem cell marker CD133 shows a slight positive correlation with the HES1 gene in colon cancer [39]. We investigated HES1 expression in the putative stem-like cells, and found that its expression increased (Figure 5A, 5B), suggesting that CD133^+^ cells display increased HES1 expression in lung cancer cells (Figure 5B).

The NOTCH signaling pathway is activated by cell-cell contact involving ligand-receptor binding. NOTCH receptors contain two domains: the extracellular domain (ECD) and the intracellular domain (ICD), and the ICD can be catalyzed by γ-secretase [40]. DAPT is a γ-secretase inhibitor and can inhibit the function of NOTCH receptors. To block the NOTCH signaling pathway, DAPT was used to pretreat the A549 cells, which decreased HES1 protein levels (Figure 5B). In contrast, DAPT had little effect on CD133 protein expression (Figure 5B, 5C).

We created an approach using five small molecule (VPA sodium salt, CHIR99021, E-616452, tranylcypromine and DZnep) compounds to stimulate A549 cells into become putative A549 stem cells *in vitro*. Other small molecules may also have the capability to reprogram A549 cells, which we plan to further investigate. Other types of lung cancer cells, such as H460 cells, also exhibited ABCG2 protein stimulation by 83.7% when treated by these compounds (Supplementary Figure 2). Further research is required to determine the optimal concentrations for these compounds and to provide a potential therapeutic target for use in the treatment of lung cancer patients.

## MATERIALS AND METHODS

### Cell culture and induction

The human NSCLC cell line A549 was purchased from the Chinese Academy of Science (Shanghai, China). A549 cells were cultured with DMEM high-glucose medium (Hyclone, Green Bay, WI, USA) supplemented with 10% fetal bovine serum (Gibco, Gaithersburg, MD USA). Cells were incubated at 37°C with 5% CO_2_ (Thermo Fisher, Waltham, MA, USA). When the cells reached about 80% confluence in a culture flask, they were digested with 0.25% trypsin (Gibco) after which 1×10^4^ A549 cells per well were seeded into 6-well plates. After the cells attached for 48 h, the culture medium was changed to DMEM/F12 medium (Hyclone) supplemented with B27 (Gibco) and two growth factors, epidermal growth factor (20 ng/ml; Shanghai Weike Biotechnology, Shanghai, China) and basic fibroblast growth factor (20 ng/ml; Shanghai Weike Biotechnology). The DMEM/F12 medium contained VPA sodium salt (1 mM; P4543, Sigma, St. Louis, MO, USA), CHIR99021 (9 μM; Cayman, Ann Arbor, MI, USA), E-616452 (25 μM; Tocris, Pittsburgh, PA, USA), Tranylcypromine (60 μM; Cayman), and DZnep (0.2 μM; Cayman). A total of 2 mL medium per well was added to each 6-well plate. The cells were induced by the five agents for 7 days. A large number of cells were apoptotic, but a small number of cells remained. We hypothesized that these cells would display stem-cell characteristics, so we called them putative A549 stem cells. To analyze the mechanism of putative A549 stem cell signaling, we used the γ-secretase inhibitor DAPT (Cayman) to inhibit the NOTCH signaling pathway. A total of 1×10^4^ A549 cells per well were seeded into 6-well plates and 2 mL DMEM/F12 medium containing 10 μM DAPT was added to every well after the cells attached for 48 h. After a 1 h incubation, the medium was changed to DMEM/F12 culture medium containing all five compounds, and left for 7 days.

### Chemotherapy resistance assay

The putative A549 stem cells were harvested, and 1×10^4^ cells per well were placed in 96-well plates. After the cells were cultured in an incubator for 48 h, different concentrations of etoposide (75, 100, 125, 150 and 200 μM) (Sigma) and cisplatin (25, 50, 100, 150 and 200 μM) (Sigma) were added to four wells with 200 μL DMEM/F12 culture medium supplemented with 10% B27. Then the cells were cultured for 9 days. For detection, the medium was changed and 10 μL Cell Counting Kit-8 (CCK8) reagent (Dojindo, Japan) with 100 μL culture medium were added to each well. The plates were cultured in an incubator at 37°C for 2 h, after which the absorption was measured at 450 nm (Bio-Tek, Winooski, VT, USA).

### Flow cytometry

To analyze the apoptotic nature of the putative A549 stem cells, the Annexin V-PE Apoptosis Detection Kit I (559763; BD Biosciences, San Jose, CA, USA) was used. A total of 5 μL Annexin V-PE and 5 μL 7-AAD in 100 μL binding buffer were incubated with 1×10^6^ cells for 15 min at room temperature in the dark. Then the cells were washed twice with phosphate-buffered saline (PBS) and resuspended with 400 μL binding buffer. Flow cytometry was used for detection (Accuri C6, BD Biosciences). To analyze the relative CD133, ABCG2, OCT4, and SOX2 gene expression in the putative A549 stem cells, the cells were incubated with 100 μL PBS containing 5 μL CD133-PE (Miltenyi Biotec, Teterow, Germany) and 2.5 μL ABCG2-PE (Miltenyi Biotec) antibodies for 30 min in the dark. Then the cells were permeabilized in cold methanol and incubated with 2.5 μL OCT4-PE (eBioscience, San Diego, CA, USA) and 2.5 μL SOX2-FITC (eBioscience) antibodies for 30 min in the dark. The cells were washed twice with PBS and resuspended with 200 μL PBS for measurement by flow cytometry.

### Quantitative real-time polymerase chain reactions

Total RNA isolation of the putative A549 stem cells and A549 cells were conducted using an RNeasy Plus Mini Kit (74136; QIAGEN, Hilden Germany). RNA concentrations were measured using a spectrophotometer (One Drop, OD-1000, China). The RNA was used to synthesize reverse transcription cDNA using a Bio-Rad script cDNA synthesis kit (Bio-Rad, Hercules, CA, USA). A total of 1 μg cDNA was used to synthesize various genes. The semi-quantitative reverse transcription polymerase chain reaction (sqRT-PCR) for each gene was conducted at an annealing temperature of 55°C. The results were analyzed using Image J software. Quantitative real-time PCR (qRT-PCR) was performed using a Mx3000P qPCR system (Stratagene, San Diego, CA, USA). A SYBR-Premix Ex Tag II (Takara, Tokyo, Japan) was used and 20 μL per gene were analyzed. The PCR for each gene was conducted at 95°C for 30 s and at 95°C for 5 s, and annealing was performed at 60°C for 34 s for 40 cycles. The results were normalized to the GAPDH gene.

### Cell invasion assay

To investigate cell invasion, 20 μL matrigel (BD Biosciences) and 80 μL DMEM/F12 medium were added to the upper layer of the transwell chamber (Corning Inc., Corning, NY, USA). A total of 1×10^4^ putative A549 stem cells were gently added to the matrigel medium, while 600 μL DMEM medium supplemented with 20% FBS was added to the chamber's lower layer. The chamber was placed in an incubator at 37°C for 48 h. Then the chamber was stained with 0.5% crystal violet in liquid methanol and incubated at 37°C for 30 min. The chamber was washed with PBS medium several times. A cotton swab was gently used to remove the cells in the upper layer of the chamber. The cells in the lower chamber were counted and statistically analyzed.

### Western blot analysis

For Western blot analysis, samples of the A549 cells induced by the small molecule chemical compounds were harvested, lysed in 200 μL RIPA lysis buffer (P0013B Beyotime, Shanghai, China), and centrifuged at 12000 rpm for 3 min. The cell lysate protein was quantified using the BCA Protein Assay Kit (Thermo Fisher). Then 15 or 20 μg of total protein sample were resolved on a 10% acrylamide gel for SDS-polyacrylamide gel electrophoresis and electrotransferred onto a PVDF membrane (Millipore, Darmstadt, Germany) at 150 mA for 120 min (Bio-Rad). Anti-CD133 (1:1000, GeneTex, Irvine, CA, USA), anti-vimentin (1:1000, Cell Signaling Technology, Danvers, MA, USA), anti-β-catenin (1:1000, Cell Signaling Technology), anti-E-cadherin (1:1000, Cell Signaling Technology), anti-NOTCH1 (1:1000, Cell Signaling Technology), anti-NOTCH2 (1:1000, Cell Signaling Technology), anti-NOTCH3 (1:1000, Cell Signaling Technology), anti-HES1 (1:1000, Abcam, Cambridge, UK), GAPDH antibody (1:10000, Abcam), and horse radish peroxidase-conjugated goat-anti-rabbit IgG (1:1000, Cell Signaling Technology) antibodies were used. Pierce ECL liquid (Thermo Fisher) was used for exposure, which was performed using a gel imaging system (Bio-Rad).

### Statistical analysis

All of the experiments were repeated three times. The statistical analyses were performed using GraphPad Prism 5 software. Comparisons between values were performed using an unpaired Student's *t* test. For all statistical analyses, the level of significance was set at a probability of <0.05 (*, P< 0.05; **, P< 0.01; ***, P< 0.001)

## SUPPLEMENTARY FIGURES


