# Supplementary material for: A combination of valproic acid sodium salt, CHIR99021, E-616452, tranylcypromine, and 3-Deazaneplanocin A causes stem cell-like characteristics in cancer cells
Source: Oncotarget. 2017 Jun 7;8(32):53302–12. doi: 10.18632/oncotarget.18396 (PMC5581111; doi:10.18632/oncotarget.18396)
Supplement: Supplementary file 1 [file oncotarget-08-53302-s001.pdf]

A combination of valproic acid sodium salt, CHIR99021, E-616452, tranylcypromine, and 3-Deazaneplanocin A causes stem cell-like characteristics in cancer cells

SUPPLEMENTARY MATERIALS

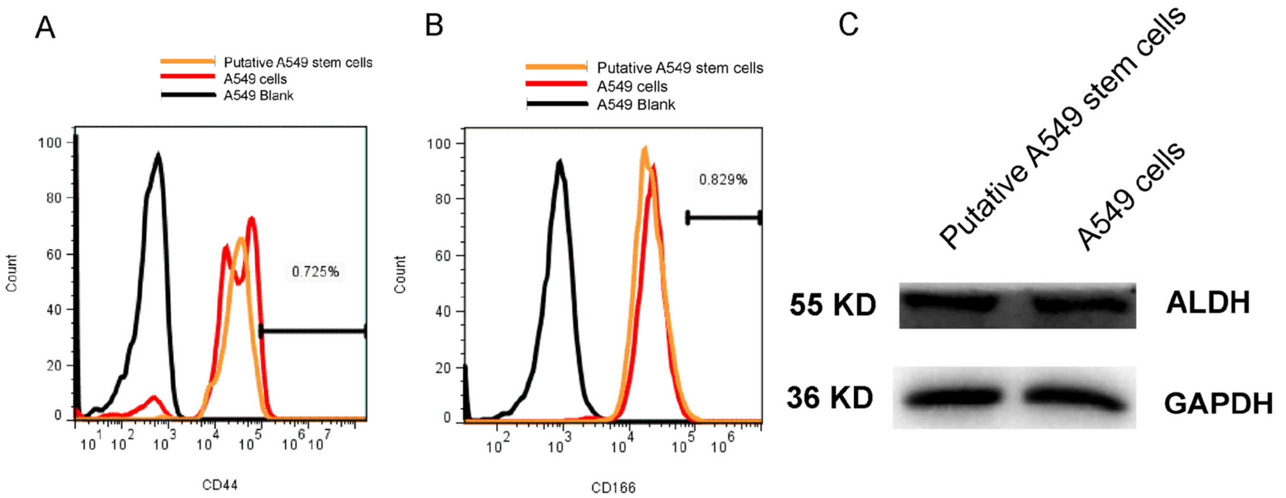

Supplementary Figure 1: Relative protein expression of putative A549 stem cells and A549 cells. **(A)** CD44 protein expression in putative A549 stem cells and A549 cells examined with flow cytometry. **(B)** CD166 protein expression in putative A549 stem cells and A549 cells examined with flow cytometry. **(C)** Western blot result for ALDH protein in putative A549 stem cells and A549 cells.

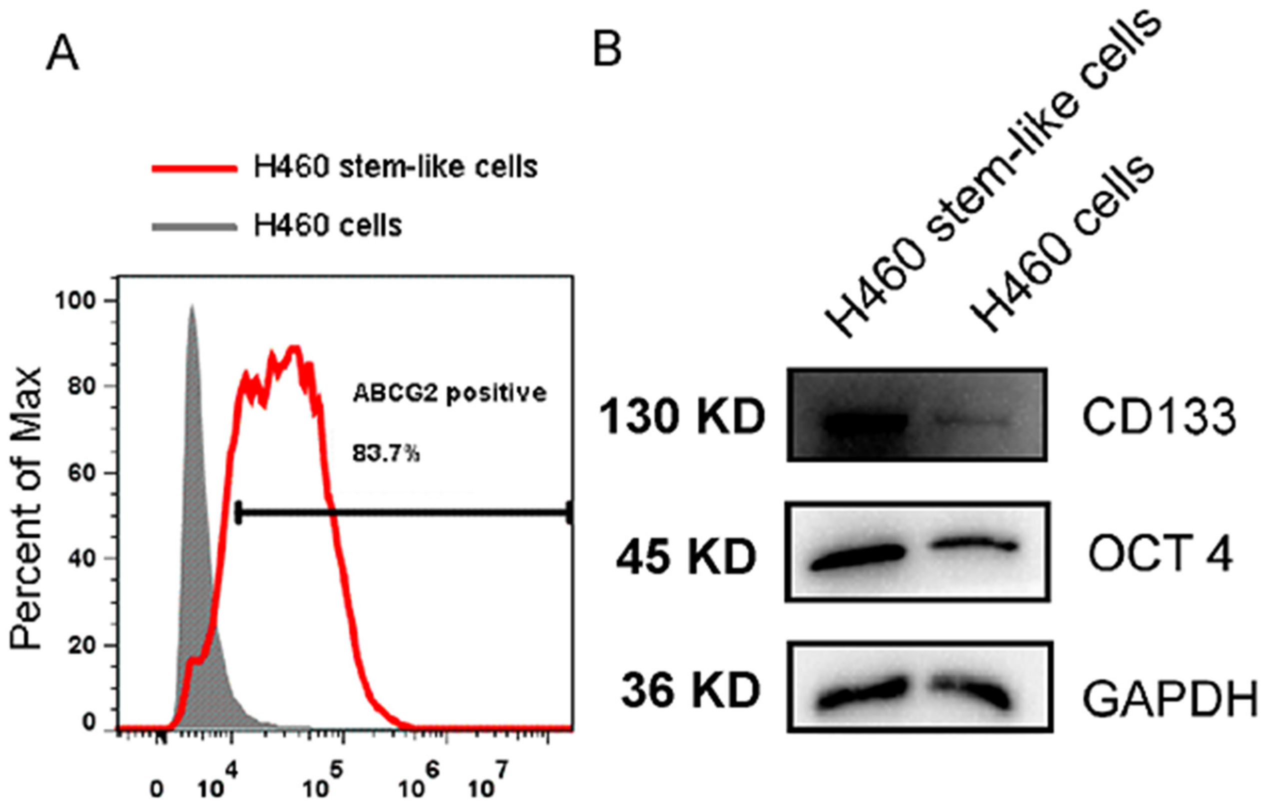

Supplementary Figure 2: Relative stem-like features of H460 stem-like cells and H460 cells. **(A)** ABCG2 protein expression in H460 stem-like cells and H460 cells examined with flow cytometry. **(B)** Western blot results for CD133 and OCT4 expression levels in H460 stem-like cells and H460 cells.
